# Prognostic accuracy of oxygen debt for mortality in patients undergoing venoarterial extracorporeal membrane oxygenation therapy: a retrospective cohort study

**DOI:** 10.3389/fmed.2025.1651531

**Published:** 2025-11-24

**Authors:** Michel Perez-Garzon, Henry Robayo-Amortegui, Angie Ochoa-Ricardo, Alejandro Quintero-Altare, Claudia Poveda-Henao

**Affiliations:** 1 Extracorporeal Life Support Unit, Department of Critical Care Medicine, Fundación Clínica Shaio, Bogotá, Colombia; 2 Critical Medicine and Intensive Care, Msc Mechanical Ventilation and Respiratory Support, Department of Investigation, Fundación Clínica Shaio, Bogotá, Colombia; 3 Facultad de Medicina, Doctorado en Ciencias Clínicas, Universidad de La Sabana, Chía, Cundinamarca, Colombia; 4 School of Medicine, Universidad de La Sabana, Chía, Cundinamarca, Colombia; 5 Department of Medicine, Critical Care Resident, Universidad de La Sabana, Chía, Cundinamarca, Colombia; 6 Clinical Cardiology, Intensive Care Department, Fundación Clínica Shaio, Bogotá, Colombia

**Keywords:** VA-ECMO, oxygen debt, mortality, intensive care, SOFA, APACHE II

## Abstract

**Background:**

Cardiogenic shock is associated with high mortality. Prognostic scales, such as Sequential Organ Failure Assessment (SOFA), Acute Physiology and Chronic Health Evaluation II (APACHE II), and Survival After Venoarterial ECMO (SAVE), have been used to estimate mortality risk or survival probability. However, their performance remains limited in the context of Venoarterial Extracorporeal Membrane Oxygenation (VA-ECMO) therapy. This study aimed to validate oxygen debt (DEOx) as a predictor of 28-day mortality in critically ill patients receiving VA-ECMO and to compare its prognostic accuracy with that of the SAVE, SOFA, and APACHE II scores.

**Methods:**

This retrospective cohort study included patients with cardiogenic shock admitted to the intensive care unit. All patients were prescribed VA-ECMO therapy in accordance with criteria by the Extracorporeal Life Support Organization. Upon initiation of ECMO, the APACHE II, SOFA, and SAVE scores, calculated 6 h prior to cannulation, and the DEOx score were compared for their predictive ability for 28-day mortality.

**Results:**

A total of 157 patients were included, with a mortality of 40% (63/157). Of these, 56.7% (89/157) were male. Mean DEOx was 11.4 mL O₂/kg. Mean age was 46.6 years (standard deviation 13.8). In multivariate analysis, variables independently associated with 28-day mortality included DEOx (odds ratio [OR]: 1.04; 95% confidence interval [CI]: 1.01–1.06; *p* = 0.001), pre-ECMO infection (OR: 2.86; 95% CI: 1.20–6.80; *p* = 0.018), hypertension (OR: 2.66; 95% CI: 1.22–5.78; *p* = 0.014), and APACHE II (OR: 1.08; 95% CI: 1.01–1.16; *p* = 0.018). Area under the curve (AUC) analysis revealed weak discrimination and similar performance regarding the primary outcome. DEOx showed the highest discrimination (AUC 0.663, 95% CI 0.49–0.77), followed by SAVE transformed to mortality (0.625), APACHE II (0.611), and SOFA (0.595).

**Conclusion:**

In adults receiving VA-ECMO for refractory cardiogenic shock, DEOx measured 6 h before ECMO cannulation showed modest discrimination for 28-day mortality and higher specificity than SOFA and SAVE at pre-specified thresholds. These findings support DEOx as a potential complementary early risk indicator; however, we did not evaluate integrated models with existing scores. Prospective, multicentre studies should evaluate whether adding DEOx to APACHE II/SOFA/SAVE improves prognostic performance and supports earlier intervention.

## Introduction

The management of critically ill patients in an intensive care unit (ICU) focuses on ensuring adequate oxygen delivery (DO₂) to maintain a balance between oxygen delivery and consumption (VO₂). Failure to sustain this balance leads to oxygen debt (DEOx), which reflects a shift from aerobic to anaerobic metabolism and is associated with organ dysfunction and increased mortality (1, 2). Although its definition may vary, DEOx is commonly understood as the difference between expected and measured VO₂ during states of shock, representing the amount of oxygen not delivered due critically reduced DO₂ (3). Despite its strong pathophysiological basis, its clinical application remains limited. However, it offers an objective measurement that is independent of variables such as age, body surface area, or temperature, and it can be calculated using base excess (BE) and lactate levels obtained through arterial blood gas analysis (1, 4). Numerous studies have linked these parameters to adverse outcomes in conditions such as hemorrhagic shock, postoperative states, and, more recently, severe SARS-CoV-2 infection (3, 5–9).

Cardiogenic shock is a critical condition characterized by impaired DO₂ secondary to myocardial dysfunction. The estimated incidence of 408 cases per 100,000 individuals and mortality rate of approximately 37%, even with the use of circulatory support strategies (10–12). Venoarterial extracorporeal membrane oxygenation (VA-ECMO), as recommended by the Extracorporeal Life Support Organization (ELSO^®^), is employed in patients with refractory cardiogenic shock as a bridge to decision-making, recovery, heart transplantation, or the use of ventricular assist devices (10–12) according to the Society for Cardiovascular Angiography and Interventions (SCAI), mechanical support is required in 30% of patients classified as stage D or E, with mortality rates ranging from 68 to 77%, respectively. Survival in patients with VA-ECMO ranges from 29 to 63.1%, and early initiation remains a significant clinical challenge (12–16).

Several prognostic tools are currently available for assessing multiorgan dysfunction syndrome, including the Sequential Organ Failure Assessment (SOFA) (17), the Acute Physiology and Chronic Health Evaluation II (APACHE II), and Simplified Acute Physiology Score II (SAPS II) (18, 19). In addition, the Survival After Venoarterial ECMO (SAVE) score can predict in-hospital survival in patients with VA-ECMO support (20). However, each of these tools has intrinsic limitations, such as overestimating the risk in patients with multiple comorbidities or advanced age, underestimation in those with extracorporeal support, and relying on laboratory data that may not be immediately available upon ICU admission (21, 22). Furthermore, none of these scoring systems incorporates the variables used to calculate DEOx, suggesting that DEOx may provide complementary prognostic information.

Although interest in the prognostic role of DEOx is increasing, no studies to date have evaluated its predictive value in patients receiving VA-ECMO support. Therefore, this study aimed to assess the performance of DEOx—using an indirect quantitative calculation—as a predictor of 28-day mortality in ICU patients undergoing VA-ECMO therapy, and to compare its prognostic utility against the SAVE, SOFA, and APACHE II scores.

## Materials and methods

### Study type

This retrospective cohort study included patients admitted to ICU with a diagnosis of cardiogenic shock SCAI D and E (14, 23) of our facility with indications for VA-ECMO support as ascertained using the ELSO^®^ criteria (12). The participant sample was drawn from patients meeting the study criteria who were treated at the Fundación Clínica Shaio in Bogotá DC, Colombia, between 8th August, 2019 and 31st October, 2024.

### Study population

Subjects aged ≥18 years with a diagnosis of cardiogenic shock according to Ponikowski et al. and indications for VA-ECMO therapy based on the ELSO^®^ criteria were determined (12, 23). To minimize transcription bias from the clinical records, the data were reviewed by at least two different evaluators and verified at the time of transcription. Each investigator provided a personal username and password and entered the data into a specifically designed online data acquisition system. Subjects with complete clinical information in the REDCap (24) information system during the entire period of care and for whom SAVE (20), APACHE II (19), SOFA (17) and DEOx scores (1, 25) could be calculated were included. Mortality data were extracted from notifications in death records and data provided in the medical history. Patients who died within the first 6 h of VA-ECMO admission, those with unreliable arterial or venous blood gas data, terminal chronic liver or kidney failure upon admission, status epilepticus, salicylate or alcohol intoxication, diabetic ketoacidosis, pediatric populations, and pregnant women were excluded, as were patients cannulated in VV mode or those requiring a change to VAV mode.

### Study variables

Data were abstracted from admission and progress notes in the electronic medical record by ICU-trained physicians following a previously standardized protocol, and included sociodemographic characteristics, comorbidities (Charlson Index) (26), admission clinical variables, laboratory and blood gas results and APACHE II (19), SOFA (17) and SAVE (20) scores. The scores were calculated 6 h before VA-ECMO cannulation, using the worst recorded physiological values available at that time. Specifically: (1) SOFA followed the original six-organ system described by Vincent et al. (17) (respiratory, coagulation, liver, cardiovascular, central nervous system, and renal domains; PaO₂/FiO₂, platelets, bilirubin, vasopressor/MAP criteria, GCS, and creatinine/urine output); (2) APACHE II followed Knaus et al. (19), comprising 12 acute physiologic variables, age, and chronic health points; and (3) SAVE followed Schmidt et al. (20) (Survival After Veno-Arterial ECMO score; integer point system per original coefficients). Full item lists, thresholds, and scoring ranges for each system, along with the DEOx calculation (DEOx = 6.322 × lactate − 2.311 × base excess − 9.013), are provided in Supplementary Table S3.

The data used for analyses and score computation, including arterial blood gas (ABG) measurements (pH, PaO₂, and PaCO₂), lactate, and base excess (BE), were abstracted from the 6 h prior to VA-ECMO cannulation. When multiple measurements were available within this window, we used the worst value for analysis (peak lactate, most negative BE, lowest PaO₂/FiO₂, highest PaCO₂). Patients without at least one ABG in this window were excluded from analyses that required these variables.

We also recorded ICU length of stay, days on VA-ECMO, and days of invasive mechanical ventilation and vasopressor support. All abstractions were reviewed by the research team to ensure that inclusion criteria were met and to prevent inconsistencies or scoring errors.

This report adheres to the STROBE statement for cohort studies; the completed STROBE checklist is provided as Supplementary Checklist S1.

### Sample size

The sample size required was calculated using the equation proposed by Obuchowski (27) for determining the confidence intervals (CIs) in diagnostic tests, along with the validity data from the original studies of SOFA (17), APACHE II (19) and SAVE (20), which report sensitivities between 65 and 92% and specificities between 62 and 90% for predicting mortality outcomes. For a 95% CI, 90% power, mortality proportion of 40%, alpha error of 0.05 and precision of 10% with Yates correction. This resulted in a minimum requirement of 135 participants.

### Statistical analysis

Quantitative variables were summarized as mean ± standard deviation (SD) when approximately normally distributed and as median (interquartile range, IQR) when skewed or non-normal; categorical variables as counts (percentages). Distributional assumptions were evaluated using Shapiro–Wilk tests and Q–Q plots. All tests were two-sided with *α* = 0.05. A bivariate analysis compared survivors and non-survivors at 28 days. Quantitative variables were compared using Student’s *t*-test or the Mann–Whitney *U* test for variables with normal or skewed distributions, respectively. Qualitative variables were compared using the chi-square test or Fisher’s exact test as appropriate.

We performed a multivariable logistic regression of 28-day mortality, excluding subjects with missing data. Variables with *p*-values <0.20 in the bivariate analysis and those judged biologically plausible were considered (28). The strength of the correlation between each variable and the proposed outcomes was estimated as an odds ratio (OR) and adjusted OR using a logistic regression model.

In multivariable logistic regression, DEOx, APACHE II, SOFA, and SAVE were entered as continuous variables and standardized (*z*-scores); ORs reflect the change in odds of 28-day mortality per 1 SD increase. Linearity with the log-odds was evaluated using restricted cubic splines (3 knots) and the Box–Tidwell approach; no material non-linearities were detected.

Sensitivity analyses. For clinical interpretability, we also evaluated pre-specified binary thresholds (DEOx ≥3.78 mL O₂/kg; SOFA ≥6; APACHE II ≥ 12; SAVE <−2 after transforming survival to mortality). Physiologic and ABG variables were analyzed using the 24 h post-cannulation window defined in Study variables.

Using the scores obtained from APACHE II, SOFA, SAVE and DEOx corrected for different confounding variables, we calculated sensitivity, specificity, positive likelihood ratio (LR+) and negative likelihood ratio (LR−), along with the areas under the curve (AUCs) and 95% CIs, at the pre-specified thresholds as detailed in the methods (DEOx 3.78 mL O₂/kg anchored to human cohorts; SOFA, APACHE II, and SAVE per their original descriptions/validations). AUROCs were compared with the DeLong test (Bonferroni-adjusted). Analyses were performed in Stata 17.0 (StataCorp, College Station, TX, United States).

Discrimination was compared using AUROC (primary, threshold-independent). For DEOx, because no VA-ECMO–specific cut-off is standardized, we pre-specified a threshold of 3.78 mL O₂/kg, anchored to human cohorts in severe COVID-19 that linked higher oxygen debt with worse outcomes (3, 9). For comparator scores, thresholds were pre-specified from the literature: SOFA [17; VA-ECMO validation (29)], APACHE II (19), and SAVE (20; survival transformed to mortality and anchored to original risk classes). Full item definitions and sources are summarized in Supplementary Table S3.

Cut-off points used for descriptive operating characteristics in Table 1 followed the thresholds reported or implied by the original studies (APACHE II ≥ 12; SOFA ≥6; SAVE <−2 when transformed from survival to mortality) and are detailed in Supplementary Table S3.

**Table 1:** Patients’ baseline characteristics.

Characteristics	Population *n* = 157	Mortality = 63	Survival = 94	*p*-value
Age in years, mean (SD)	46.6 (13.8)	49.3 (13.7)	44.7 (13.6)	0.978
Male, *n* (%)	89 (56.7)	41 (65.1)	48 (51.1)	0.082
Body mass index, mean (SD)	25.9 (4.3)	26.4 (4.4)	25.7 (4.2)	0.821
Outpatient days – Median (IQR)	4.8 (3.8–5.9)	4.4 (3.1–5.7)	5.1 (3.6–6.6)	0.865
Pre-ECMO MV days – Median (IQR)	1.8 (1.5–2.1)	2.3 (1.6–2.9)	1.5 (1.3–1.7)	0.314
Days from admission to ECMO initiation – Median (IQR)	1.7 (1.1–2.3)	2.2 (0.9–3.4)	1.4 (0.7–2.1)	0.768
Heparin days – Median (IQR)	5.6 (4.7–6.5)	5.2 (4.2–6.1)	5.8 (4.5–7.1)	0.526
ICU days – Median (IQR)	25.8 (17.9–33.7)	14.8 (10.7–19.1)	33.5 (20.5–46.4)	0.003*
MV days – Median (IQR)	11.9 (10.2–13.6)	11.6 (8.4–14.8)	12.1 (10.3–13.9)	0.400
LVEF – Median (IQR)	33.4 (29.2–37.6)	32.4 (25.7–39.2)	34.1 (28.5–39.7)	0.349
Referral, *n* (%)	91 (57.9)	36 (57.1)	55 (58.5)	0.865
IABP counterpulsation support, *n* (%)	61 (38.8)	26 (41.3)	35 (37.2)	0.611
Pre-ECMO infection, n (%)	26 (16.6)	16 (25.4)	10 (10.6)	0.015*
Comorbidities, *n* (%)				
High blood pressure	34 (21.6)	20 (31.7)	14 (14.9)	0.012*
Pulmonary hypertension	14 (8.9)	7 (11.1)	7 (7.4)	0.430
Dyslipidaemia	17 (10.8)	5 (7.9)	12 (12.8)	0.340
Heart failure	21 (13.4)	6 (9.5)	15 (15.9)	0.246
Hypothyroidism	16 (10.2)	7 (11.1)	9 (9.6)	0.755
Diabetes mellitus 2	24 (15.3)	13 (20.6)	11 (11.7)	0.127
Atrial fibrillation	11 (7.1)	6 (9.5)	5 (5.3)	0.312
Pulmonary thromboembolism	11 (7.1)	3 (4.8)	8 (8.5)	0.367
Coronary heart disease	10 (6.4)	5 (7.9)	5 (5.3)	0.876
Smoking	12 (7.6)	6 (9.5)	6 (6.4)	0.468
Valvular heart disease	24 (15.3)	12 (19.1)	12 (12.8)	0.284
Pre-ECMO laboratory parameters, mean (SD)				
D-dimer	4637.4 (7772.2)	4485.9 (6590.9)	4749.1 (8716.9)	0.461
Lactate dehydrogenase	595.8 (408.3)	829 (557.5)	421 (176.6)	0.834
Leucocytes ×10^3^/μL	13.1 (6.7)	13 (6.8)	13.2 (6.7)	0.405
Hemoglobin g/dL	12.9 (2.7)	12.6 (2.7)	13.2 (2.6)	0.079
Platelets ×10^3^/μL	204.2 (116.5)	198.1 (133.8)	208.5 (103.3)	0.309
Thromboplastine time	36.8 (16.5)	39.3 (17.8)	35.1 (15.4)	0.905
Sodium mEq/L	138.2 (4.8)	137.8 (5.1)	138.6 (4.6)	0.181
Potassium mEq/L	4.4 (0.8)	4.3(0.8)	4.5 (0.8)	0.255
Creatinine mg/dL	1.83 (1.56)	2.15 (2.01)	1.62 (1.13)	0.966
Fibrinogen	259.2 (120.6)	244.1 (92.1)	268.7 (137.5)	0.295
Blood gasses upon admission, mean (SD)				
pH	7.30 (0.2)	7.27 (0.2)	7.31 (0.1)	0.080
HCO3 meq/L	19.1 (5.3)	19.4 (5.1)	18.9 (5.5)	0.721
Arterial CO₂ partial pressure (PaCO₂), mmHg	39.9 (14.8)	43.4 (15.2)	37.4 (14.1)	0.989
Arterial O_2_ saturation %	91.5 (7.7)	89.8 (9.1)	92.5 (6.5)	0.064
Venous O_2_ saturation %	65.6 (13.6)	64.3 (13.4)	66.6 (13.8)	0.218
Base excess	−6.2 (7.5)	−7.4 (7.2)	−5.3 (7.7)	0.061
Lactate mmol/L	4.3 (3.3)	4.0 (2.9)	4.5 (3.6)	0.221
Severity score, Median (IQR)				
SOFA Score	7 (5–8)	7 (5–9)	7 (5–8)	0.157
APACHE II Score	11 (8–14)	12 (9–16)	11 (7–14)	0.022*
DEOx, mean (SD)	11.4 (27.9)	23.6 (40.7)	4.3 (12)	0.001*
SAVE Score	1 (−2 to 3)	0 (−2 to 2)	1 (−2 to 3)	0.032*

SD, standard deviation; M, median; IQR, interquartile range; ECMO, extracorporeal membrane oxygenation; SOFA, sequential organ failure assessment; APACHE, acute physiology and chronic health disease classification system; DEOx, oxygen debt; MV, mechanical ventilation; LVEF, left ventricular ejection fraction; IABP counterpulsation, intra-aortic balloon counterpulsation; HCO3, bicarbonate; *p < 0.05.

Data are reported as mean ± SD or median (IQR), and *n* (%) for categorical variables.

## Results

**Figure 1:**
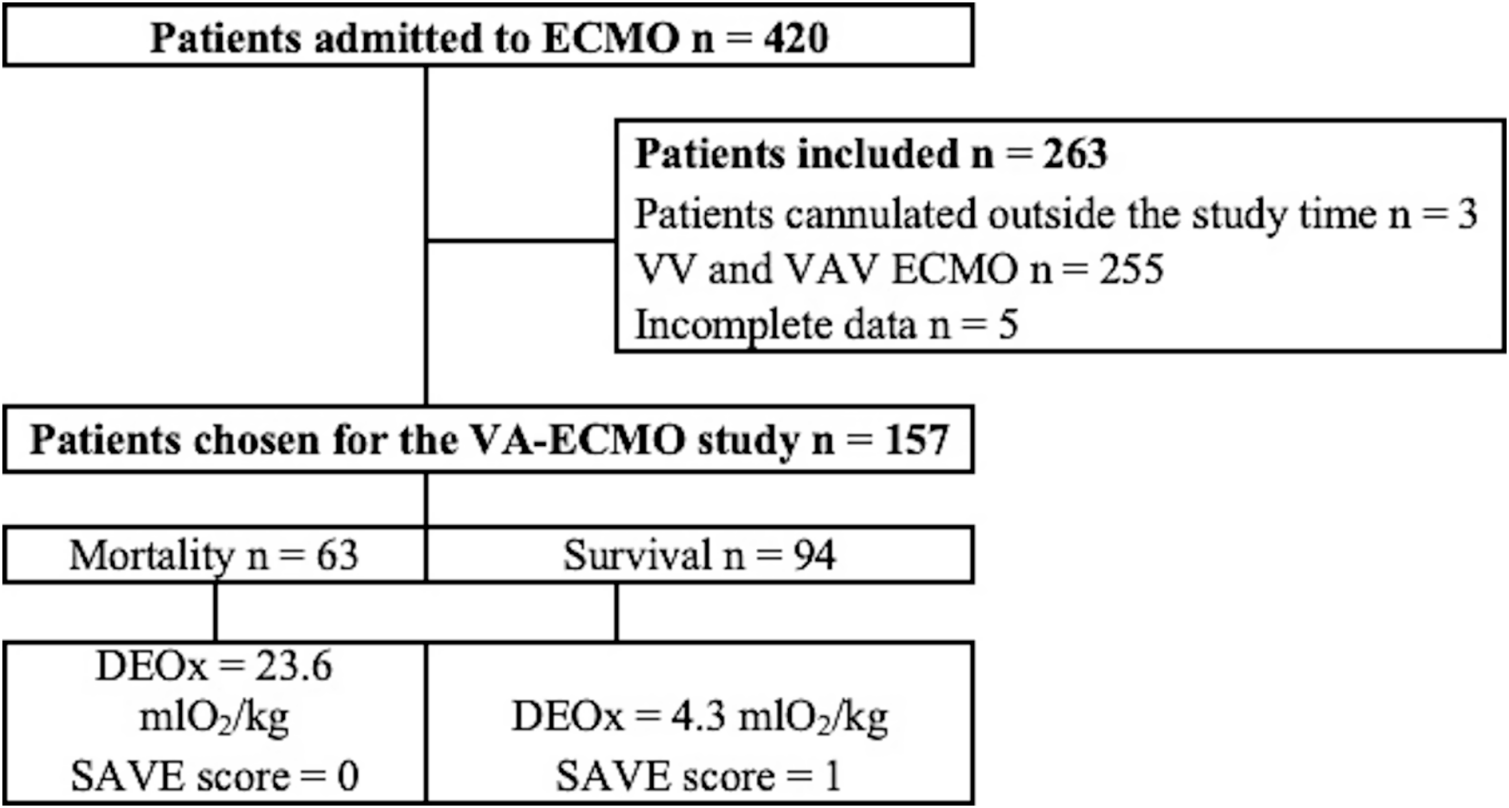
Study subject admission flowchart.

A total of 420 patients were admitted to the ICU for ECMO therapy during the study period, of which 263 patients did not meet the inclusion criteria, leaving 157 subjects for the final analysis, where 63/157 (40%) died, showing an average DEOx value of 11.4 mL O_2_/kg. Figure 1 illustrates the flow of subjects into the study.

### Study population characteristics

The mean age of the patients was 46.6 years (SD: 13.8), with 56.7% (89/157) being male. Time from admission to ECMO initiation: 1.7 days (IQR 1.1–2.3); duration of mechanical ventilation: 11.9 days (IQR 10.2–13.6) and left ventricle ejection fraction 33.4% (IQR: 29.2–37.6). The most prevalent comorbidities were arterial hypertension (21.6%), diabetes mellitus (15.3%) and valvulopathy (15.3%). A significant relationship was found between mortality and ICU length, infection before ECMO and hypertension. Table 2 summarizes the baseline characteristics of the population and their relationship with mortality. Unless otherwise specified, quantitative variables are presented as mean ± SD (normal distributions) or median (IQR) (skewed distributions).

**Table 2:** Pre-ECMO cardiovascular conditions.

Characteristics	Population *n* = 157	Mortality = 63	Survival = 94	*p*-value
Pre-ECMO, *n* (%)	40 (25.5)	20 (31.7)	20 (21.3)	0.140
Pre-ECMO surgery, *n* (%)	63 (40.4)	22 (35.5)	41 (43.6)	0.311
Pre-ECMO ECC, *n* (%)	47 (29.9)	18 (81.8)	29 (78.4)	0.751
Pump time (min), median (IQR)	183.7 (156.4–210.9)	175.2 (125.9–224.5)	188.9 (154.7–223.1)	0.318
Ischaemia time – min, median (IQR)	107.7 (91.9–123.5)	103.3 (75.4–131.3)	110.4 (90.3–130.6)	0.334
Pre-ECMO support, *n*(%)				
Noradrenaline	149 (94.9)	62 (98.4)	87 (92.5)	0.102
Vasopressin	131 (83.4)	55 (87.3)	76 (80.8)	0.287
Adrenaline	49 (31.2)	23 (36.5)	26 (27.7)	0.241
Dobutamine	77 (49.1)	31 (49.2)	46 (48.9)	0.974
Levosimendan	53 (33.8)	21 (33.3)	32 (34.1)	0.927
Milrinone	34 (21.7)	14 (22.2)	20 (21.3)	0.888
No vasopressors	26 (16.6)	12 (19.1)	14 (14.9)	0.493
Pre-ECMO support dose, mean (SD)				
Noradrenaline, mcg/kg/min	0.58 (0.52)	0.63 (0.60)	0.54 (0.45)	0.824
Vasopressin, U/h	3.71 (1.59)	3.88 (1.71)	3.59 (1.50)	0.833
Adrenaline, mcg/kg/min	0.28 (0.28)	0.28 (0.31)	0.28 (0.25)	0.489
Dobutamine, mcg/kg/min	8.4 (5.1)	8.7 (5.3)	8.2 (5.1)	0.631
Milrinone, mcg/kg/min	0.42 (0.11)	0.41 (0.07)	0.42 (0.13)	0.386

SD, standard deviation; M, median; IQR, interquartile range; ECMO, extracorporeal membrane oxygenation; ECC, extracorporeal circulation; Min, minutes; *p < 0.05.

Data are reported as mean ± SD or median (IQR), and *n* (%) for categorical variables.

Regarding pre-cannulation cardiovascular conditions, no statistically significant differences were found between patients who survived and those who died. Although cardiac arrest before ECMO and cardiopulmonary bypass use before ECMO were slightly more frequent in non-survivors, these differences did not reach statistical significance. Similarly, the use of vasoactive/inotropic support was high, with no relevant differences between groups. Norepinephrine was the most frequently used drug (94.9%), followed by vasopressin (83.4%) and adrenaline (31.2%), with no significant differences in administered doses. Table 3 summarizes the pre-cannulation cardiovascular conditions, and the Supplementary Tables S1, S2 describes the causes of cardiogenic shock and those related to surgical events.

**Table 3:** Additional characteristics related to VA-ECMO use.

Characteristics	Population *n* = 157	Mortality = 63	Survival = 94	*p*-value
Heparin use, *n* (%)	93 (59.2)	30 (47.6)	63 (67.1)	0.015*
Maximum heparin dose, mean (SD)	736.5 (310.8)	713.3 (266.2)	747.6 (331.3)	0.297
Minimum heparin dose, mean (SD)	276.3 (97.4)	283.3 (111.7)	273.1 (90.6)	0.669
ECMO flow rate at 4 h, L/min, mean (SD)	3.3 (0.9)	3.3 (0.9)	3.4 (0.8)	0.304
ECMO flow at 24 h L/min, mean (SD)	3.6 (0.9)	3.6 (0.8)	3.5 (1.0)	0.738
ECMO RPM	3,512 (923.7)	3504.6 (918.7)	3517.8 (931.9)	0.465
Transfusions, *n* (%)				
Packaged red blood cells	117 (74.5)	53 (84.1)	64 (68.1)	0.024*
Fresh frozen plasma	84 (53.5)	41 (65.1)	43 (45.7)	0.017*
Platelets	122 (77.7)	50 (79.4)	72 (76.6)	0.683
Cryoprecipitates	50 (31.8)	23 (36.5)	27 (28.7)	0.305
No transfusion	11 (7.1)	2 (3.2)	9 (9.6)	0.124
Transfusion volume mL, mean (SD)				
Packaged red blood cells	2597.5 (2395.4)	2673.7 (2085.9)	2534.5 (2639.1)	0.624
Fresh frozen plasma	1839.7 (1187)	1974.1 (1350.4)	1711.6 (1006.6)	0.841
Platelets	1908.2 (1525.1)	2187.3 (1792.1)	1714.3 (1286.2)	0.943
Cryoprecipitates	298.6 (180.7)	277.5 (146.8)	316.6 (206.3)	0.219

SD, standard deviation; ECMO, extracorporeal membrane oxygenation; RPM, revolutions per minute; *p < 0.05.

Data are reported as mean ± SD or median (IQR), and *n* (%) for categorical variables.

The prognostic scores showed statistically significant differences between patients who died and those who survived for APACHE II, SAVE score and DEOx. Additionally, red blood cell (RBC) transfusions were significantly more frequent in non-survivors (84.1% vs. 68.1% in survivors, *p* = 0.024), the use of fresh frozen plasma was higher among patients who died (65.1% vs. 45.7%, *p* = 0.017) and heparin use was significantly higher among survivors (67.1%) than among non-survivors (47.6%) (*p* = 0.015). Table 4 summarizes the additional clinical conditions associated with mortality.

**Table 4:** Factors associated with mortality in VA-ECMO.

Outcome	OR (95% CI)	*p*-value	aOR (95% CI)	*p*-value
Age	1.03 (0.99–1.06)	0.053*	1.02 (1.01–1.04)	0.044*
Arterial hypertension	2.43 (0.86–6.82)	0.093	2.66 (1.22–5.78)	0.014*
Pre-ECMO haemoglobin	0.88 (0.74–1,03)	0.118	0.91 (0.79–1.03)	0.154
Pre-ECMO noradrenaline	2.48 (0.20–30.6)	0.477	4.9 (0.59–41.57)	0.137
Pre-ECMO Infection	3.35 (1.03–10.87)	0.043*	2.86 (1.20–6.80)	0.018*
DEOx score	1.06 (1.02–1.09)	0.001*	1.04 (1.01–1.06)	0.001*
APACHE II Score	1.08 (0.98–1.18)	0.110	1.08 (1.01–1.16)	0.018*

OR, odds ratio; aOR, adjusted odds ratio; CI, confidence interval; ECMO, extracorporeal membrane oxygenation; APACHE, acute physiology and chronic health disease classification system; DEOx, oxygen debt; *p < 0.05.

### Multivariate analysis

DEOx demonstrated a statistically significant association as an independent variable for 28-day mortality. The variables with the highest OR were infection before ECMO with an OR of 2.86 (95% CI: 1.20–6.80; *p* = 0.018), hypertension with an OR of 2.66 (95% CI: 1.22–5.78; *p* = 0.014), APACHE II score with an OR of 1.08 (95% CI: 1.01–1.16; *p* = 0.018) and DEOx with an OR of 1.04 (95% CI: 1.01–1.06, *p* = 0.001). In the multivariate analysis, the variables independently associated with the studied outcomes are presented in Tables 5, 6.

**Table 5:** Factors associated with 28-day mortality in VA-ECMO (multivariable logistic regression; predictors entered as standardized z-scores).

Outcome	OR (95% CI)	*p*-value	aOR (95% CI)	*p*-value
SOFA Score	1.09 (0.97–1.22)	0.136	1.04 (0.90–1.20)	0.563
APACHE II score	1.08 (1.01–1.16)	0.018*	1.04 (0.96–1.13)	0.121
SAVE score	0.90 (0.82–0.99)	0.040*	0.91 (0.82–1.02)	0.125
High DEOx	2.52 (1.28–4.94)	0.007*	2.53 (1.20–5.32)	0.014*

OR, odds ratio; aOR, adjusted odds ratio; CI, confidence interval; SOFA, sequential organ failure assessment; DEOx, oxygen debt; ECMO, extracorporeal membrane oxygenation; APACHE, acute physiology and chronic health disease classification system; SAVE, survival after venoarterial ECMO; *p < 0.05.

ORs represent the change in odds of 28-day mortality per 1 SD increase in each continuous predictor.

**Table 6:** Prediction of mortality by prognostic scores for VA-ECMO.

Score	Cut-off point	Se	Sp	LR+	LR−	AUROC (95%CI)
APACHE II	12	54.1%	55.2%	1.21	0.83	0.611 (0.51–0.71)
SOFA	6	63.9%	38.6%	1.04	0.93	0.595 (0.49–0.69)
SAVE score	<−2	75.4%	20.2%	0.94	1.21	0.375 (0.27–0.47)
DEOx	3.78	64.7%	56.3%	1.48	0.63	0.663 (0.49–0.77)
Lactate	>2	75.9%	31.5%	1.11	0.76	0.501 (0.41–0.593)
Base excess	<−2	26.9%	72.6%	0.98	1.01	0.447 (0.36–0.54)
	<−5	42.3%	57.5%	0.99	1.01	0.447 (0.36–0.54)

Se, sensitivity; Sp, specificity; LR+, positive likelihood ratio; LR−, negative likelihood ratio; AUROC, area under the receiver operating characteristic curve; CI, confidence interval. DEOx metrics were calculated at a pre-specified threshold of 3.78 mL O₂/kg (anchored to human cohorts), whereas thresholds for SOFA, APACHE II, and SAVE were pre-specified from their original descriptions/validations (see Methods and Supplementary Table S3).

SAVE AUROC corresponds to survival; for mortality orientation AUROC = 0.625.

### 28-day mortality performance of APACHE II, SOFA, SAVE, and DEOx

**Figure 2:**
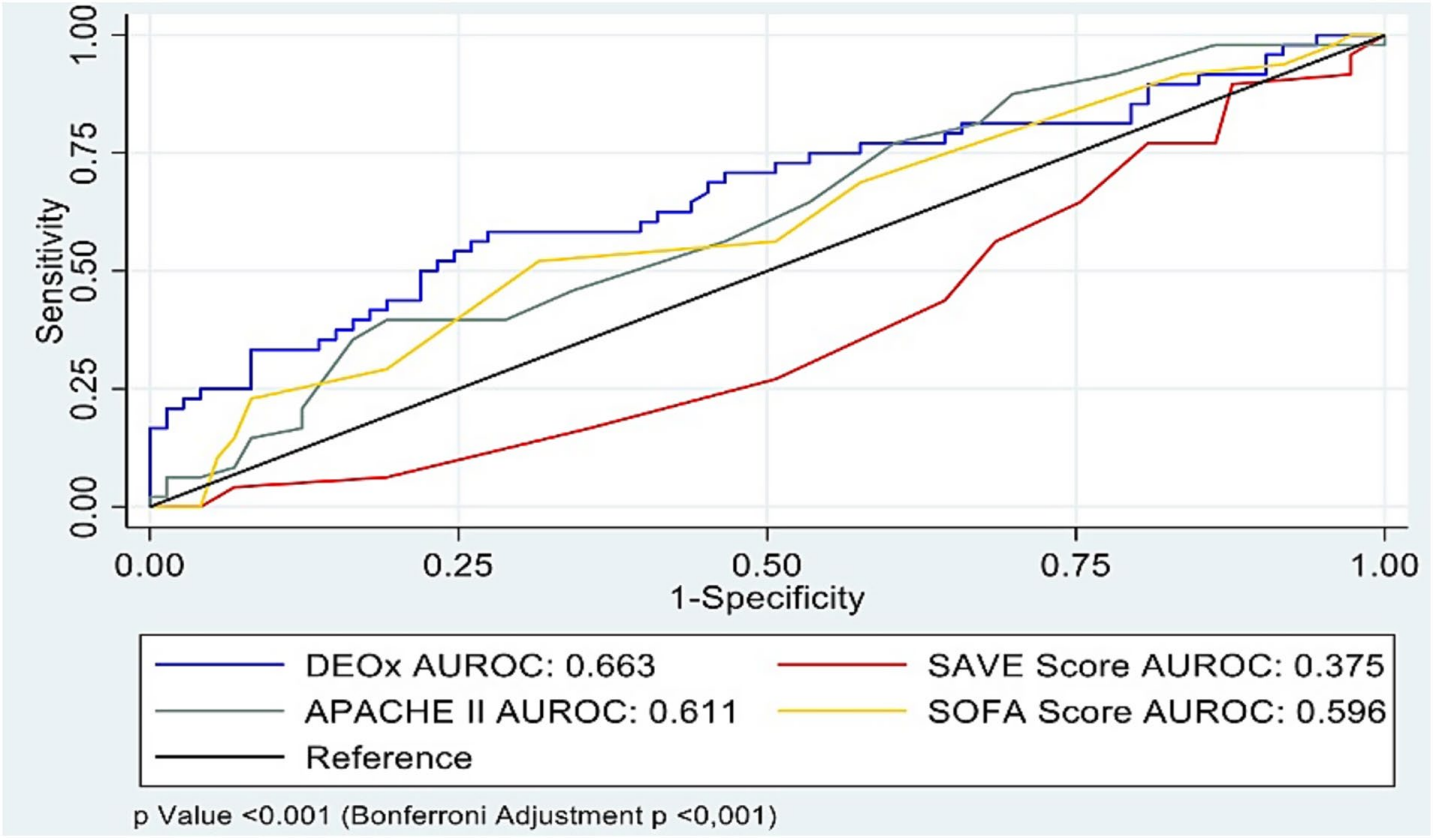
DEOx vs. 28-day mortality in VA-ECMO.

For 28-day mortality, the AUROC values were 0.663 for DEOx (95% CI 0.49–0.77), 0.611 for APACHE II (95% CI 0.51–0.71), 0.595 for SOFA (95% CI 0.49–0.69), and 0.625 for SAVE after reversing its orientation from survival to mortality calculated as 1-survival (untransformed AUROC for survival 0.375, 95% CI 0.27–0.47). Pairwise DeLong comparisons showed overall differences across curves (*p* < 0.001; Bonferroni-adjusted *p* < 0.001). AUROC estimates for all four tools are summarized in Table 1 and displayed in Figure 2.

At the pre-specified cut-offs, SAVE had the highest sensitivity (75.4%; cut-off < −2), whereas DEOx provided the highest specificity (56.3% at 3.78 mL O₂/kg). Operating characteristics for DEOx in Table 1 were computed at the pre-specified threshold of 3.78 mL O₂/kg (anchored to human cohorts), while thresholds for SOFA, APACHE II, and SAVE were pre-specified from their original descriptions/validations. Threshold-based operating characteristics (sensitivity, specificity, LR+, LR−) for each score are reported in Table 1; for item-level composition and operational definitions of each score (see Supplementary Table S3).

## Discussion

This study is the first to evaluate DEOx as a predictor of 28-day mortality among patients undergoing VA-ECMO support. Although the predictive capacity of DEOx as a single variable was limited, it outperformed the SOFA score and was comparable to established tools such as APACHE II and SAVE, all of which were calculated 6 h prior to ECMO cannulation, with higher specificity for mortality prediction. Several reports have described higher AUROC values for the SAVE (20), SOFA (29), and APACHE II scores (30) —frequently exceeding 0.8—when predicting mortality in VA-ECMO patients. In contrast, all scores in our study exhibited lower discrimination. This discrepancy may be attributed to differences in patient characteristics and timing of score assessment. In our cohort, pre-ECMO infection was present in 16.6% of patients and hypertension in 21.6%, both independently associated with increased mortality. To assess a pre-cannulation state, all scores were calculated 6 h prior to ECMO cannulation, a period that still reflects the patient’s condition before the pronounced hemodynamic and metabolic instability of the initial post-cannulation phase (20, 29). Finally, as a single-center retrospective study, site-specific practices, including center volume, may have influenced observed performance (31, 32). These considerations underscore the need to contextualize prognostic score performance and support the complementary value of DEOx, particularly for early (<24 h) risk stratification. A modified version of the SAVE score, proposed by Santore et al. (59) integrates pre-ECMO lactate and bicarbonate values to improve predictive accuracy for in-hospital mortality. Although not included in our comparative analysis due to limited access to the exact scoring algorithm, this approach conceptually supports the inclusion of metabolic parameters, such as those used in the DEOx calculation as prognostic indicators. Given that DEOx incorporates both lactate and base excess, our findings align with the physiological rationale underpinning this modified score. Future studies should compare DEOx directly against this and other metabolically enriched prognostic models to assess incremental predictive value (59).

In our cohort, discrimination for 28-day mortality was modest across all four tools—AUROC 0.663 for DEOx (95% CI 0.49–0.77), 0.611 for APACHE II (95% CI 0.51–0.71), 0.595 for SOFA (95% CI 0.49–0.69), and 0.625 for SAVE after transforming survival to mortality; pairwise comparison with the DeLong test (Bonferroni-adjusted) indicated overall differences across curves (*p* < 0.001). Operating characteristics diverged: DEOx provided the highest specificity (56.3%) and the most favorable LR + 1.48 and LR − 0.63, whereas SAVE yielded the highest sensitivity (75.4%) but very low specificity (20.2%) and suboptimal likelihood ratios at the predefined threshold; SOFA and APACHE II showed intermediate, more balanced profiles. These operating points correspond to the values reported in Table 1. These findings are consistent with the constructs each score captures: DEOx reflects early metabolic debt (lactate and base excess), SOFA concurrent organ dysfunction, APACHE II acute physiologic derangement plus chronic health status, and SAVE modeled survival probability (transformed here to mortality). All scores were calculated within the first 24 h post-cannulation, a phase of hemodynamic instability that may attenuate discrimination relative to later assessments. Clinically, this pattern supports a complementary strategy: DEOx as a rule-in aid for high-risk identification, with APACHE II/SOFA providing broader physiologic context, and SAVE offering sensitivity but limited specificity; thus, DEOx should complement rather than replace established scores.

Perez-Garzon et al. (3) reported a difference of 3.37 mL O_2_/kg DEOx between survivors and non-survivors in patients with SARS-CoV-2 infection, suggesting that higher DEOx values suggest a higher risk of mortality, while in our cohort this difference was higher, reaching 19.3 mL O_2_/kg. Similarly, in human cohorts with severe COVID-19, DEOx values around 3.78 mL O₂/kg have been reported in association with metabolic derangement and worse outcomes (3, 9), processes that are central to the pathophysiology of cardiogenic shock (33–35) Shoemaker et al. (6) measured DEOx in 100 high-risk post-surgical patients and found that non-survivors had a cumulative deficit of 26.8 ± 32.1 L/m^2^ compared with survivors (8.0 ± 10.9 L/m^2^). Beyond its role in mortality prediction, DEOx has been proposed as a predictor of the need for orotracheal intubation in patients treated with high-flow nasal cannulas and as a marker of acute intestinal injury in SARS-CoV-2 (8, 9). The study by Kurniawati et al. (36) found that correction of DEOx within the first 24 h after ECMO support initiation was positively correlated with survival. These findings underscore that not only the magnitude of accumulated DEOx is clinically relevant but also the capacity to rapidly restore efficient aerobic metabolism—highlighting the importance of minimizing the duration of oxygen debt to improve patient outcomes (37); in the present VA-ECMO cohort this value is considered only as a sensitivity benchmark, whereas our primary analysis relies on threshold-independent discrimination and a cohort-optimized Youden cut-off.

The prognostic value of lactate and BE have been recognized as marker of severity, clinical progression and mortality in critically ill patients (36–39). In cardiogenic shock requiring mechanical circulatory support, higher admission lactate and lower 24-h lactate clearance were independently associated with increased mortality in external cohorts (40). Consistently, higher lactate at 24 h has also been linked to worse outcomes (41). By integrating lactate and BE into a single construct, DEOx may better reflect the transition to anaerobic metabolism and its impact on survival (36). In addition, Smuszkiewicz et al. (42) reported that BE <−9.5 mmol/L was associated with a four-fold increase in mortality risk (adjusted hazard ratio: 4.22; 95% CI: 2.21–8.05; *p* < 0.0001). Rajsic et al. (43) conducted a meta-analysis of 32 studies involving 12,756 adults on VA-ECMO, and identified infection (including sepsis and ICU-acquired pneumonia) as associated with higher in-hospital mortality (*p* = 0.017). Similarly, Vogel et al. (44) reported that patients with pre-ECMO bacteremia were at greater risk of infectious complications (OR: 2.12; 95% CI: 1.92–2.34; *p* < 0.001), contributing to worse clinical outcomes. Fernando et al. (45), in their retrospective cohort study of 15,172 patients on VA-ECMO, reported age >40 years (OR: 1.26, 95% CI: 1.08–1.47) as an independent mortality factor and Hashem et al. (46) showed in a meta-analysis of 931 patients that age >65 years predicted increased mortality (OR: 4.61, 95% CI: 1.63–13.03, *p* < 0.01). In a cohort of 312 patients who were weaned from support, they found that, compared to survivors, non-survivors were older (66.6 ± 14.0 vs. 58.7 ± 13.8 years; *p* < 0.001) and had a higher prevalence of comorbidities, including hypertension (62.5% vs. 40.2%; *p* = 0.005), diabetes mellitus (56.3% vs. 33.6%; *p* = 0.006), dyslipidaemia (41.7% vs. 18.2%, *p* = 0.003) and chronic kidney disease (14.5% vs. 3.7%; *p* < 0.001) (8). In the logistic regression analysis, systemic arterial hypertension was independently associated with in-hospital mortality (inverse association; OR: 0.40; 95% CI: 0.211–0.768; *p* < 0.006) (47). Similarly, Vigneshwar et al. (39) found higher in-hospital mortality in VA-ECMO patients with systemic arterial hypertension (41.6% vs. 33.4%; *p* = 0.02).

Regarding transfusions, Deatrick et al. (38) reported a 3% increase in mortality per unit of RBCs transfused in adults on ECMO after multivariable adjustment (OR of 1.03, 95% CI: 1.00–1.06, *p* = 0.04). Quin et al. (48) documented transfusion of blood products as an independent risk factor for mortality (adjusted OR: 1.09; 95% CI: 1.01–1.18; *p* = 0.035), and Guimbretière et al. (49) observed a mortality > 80% in patients with high transfusion requirements (≥19 units of RBCs, ≥5 units of platelets, or ≥12 units of FFP). Similarly, Li et al. (50), confirmed in a meta-analysis of 8 studies (*n* = 794) that higher total RBC volumes were significantly associated with increased mortality (Standardized weighted difference 0.62; 95% CI: 1.06–0.18; *p* = 0.006; I2 = 79.7%; p-heterogeneity = 0.001), supporting a restrictive transfusion strategy in critically ill patients without an optimal threshold, especially VA-ECMO (51, 52). Luo et al. (53) reported FFP transfusion as an independent factor for in-hospital ECMO mortality (OR 1.09, 95% CI: 1.01–1.18; *p* = 0.035). These findings are consistent with ours, in which a higher transfusion rate were documented in non-survivors, aligning with recommendations against prophylactic hemocomponent use (54).

The ELSO^®^ 2022 registry reported a higher incidence of thrombotic and hemorrhagic complications in VA-ECMO compared with VV-ECMO. The rate of circuit thrombosis was 0.225 per 1,000 h of support, whereas the rate of major thrombotic events, such as cerebral infarction, was 0.208/1,000 h. Hemorrhagic complications were more frequent (0.871/1,000 h), highlighting the need for anticoagulation with strict monitoring (43). Lv et al. (55) showed in a meta-analysis (7 studies, *n* = 553) that low-dose unfractionated heparin (aPTT 40–60 s) was associated with a significant reduction in bleeding—especially gastrointestinal (OR 0.36, 95% CI 0.20–0.64)—and surgical-site bleeding (OR 0.43, 95% CI 0.20–0.94), without increasing thrombotic complications, ECMO withdrawal, or mortality (OR 0.81, 95% CI 0.42–1.56). Garzón-Ruiz et al. (56) reported that targeting PTT < 60 s was protective, with a 30% reduction in bleeding for PTT 50–60 s, 22% for PTT 40–50 s, and 47% for PTT ≤ 40 s, without differences in thrombotic events. These observations are consistent with our data, which showed improved survival associated with heparin use.

We did not derive or validate a composite model integrating DEOx with established scores; therefore, any potential gain from combining DEOx with APACHE II, SOFA, or SAVE remains to be tested in prospective, multicentre cohorts.

Our study has limitations inherent in its retrospective design. First, there is a risk of information bias, as the analysis depends on the quality of clinical records. However, the institution has a concurrent data-collection system staffed by clinicians trained by the research group (57). Second, being a single-center study, extrapolation of results may be limited, although internal validity is supported by the standardized methodology, data cross-verification, multiple analysis approaches, and ELSO^®^ Gold-Center certification. Third, DEOx estimation depends on the quality and timing of arterial blood gas factors we addressed through predefined exclusion criteria and standardized timing. Moreover, we analyzed a single DEOx value without incorporating temporal trajectories, which may offer additional prognostic insight in future studies. Lastly, while DEOx was compared with validated systems such as APACHE II and SOFA, these scores also have known limitations in ECMO populations, and the lack of an external validation cohort restricts generalizability. Future multicenter prospective studies are essential to establish DEOx as a validated prognostic tool in VA-ECMO therapy.

## Conclusion

The DEOx as a predictor of 28-day mortality in critically ill patients with cardiogenic shock indicated for VA-ECMO therapy was similar to APACHE II and SAVE, and superior to the SOFA scores. The correlation of DEOx with these scales may be useful for making early interventions in critically ill patients, with easy clinical applicability at the patient’s bedside. Prospective studies are needed to evaluate its usefulness for continuous monitoring and decision-making during VA-ECMO support.

## Data Availability

The raw data supporting the conclusions of this article will be made available by the authors, without undue reservation.

## Glossary

DEOx: Oxygen Debt
ECLSU: Extracorporeal Life Support Unit
ICU: Intensive Care Unit
DO2: Oxygen Delivery
VO2: Oxygen Consumption
VA-ECMO: Extracorporeal Venoarterial Membrane Oxygenation
ELSO^®^: Extracorporeal Life Support Organization
SCAI: Society for Cardiovascular Angiography and Intervention
SOFA: Sequential Organ Failure Assessment
APACHE II: Acute Physiology and Chronic Health Evaluation II
SAVE: Survival After Venoarterial ECMO
VV: Venovenous
VAV: Venoarteriovenous
BMI: Body Mass Index
BE: Base Excess
AUC: Area Under the Curve
IQR: Interquartile Ranges
SD: Standard Deviation
OR: Odds Ratio
AUROC: Area Under Receiver Operating Characteristic
CI: Confidence Interval
RBCs: Red Blood Cells
FFP: Fresh Frozen Plasma
