# Supplementary material for: Prognostic accuracy of oxygen debt for mortality in patients undergoing venoarterial extracorporeal membrane oxygenation therapy: a retrospective cohort study
Source: Front Med (Lausanne). 2025 Nov 24;12:1651531. doi: 10.3389/fmed.2025.1651531 (PMC12682869; doi:10.3389/fmed.2025.1651531)
Supplement: Supplementary file 1 [file Data_Sheet_1.docx]

**Supplementary Material**

|  |  |  |  |  |  |  |
| --- | --- | --- | --- | --- | --- | --- |
|  | **Supplementary Table 1. Causes of cardiogenic shock** | | | | |  |
|  | **Characteristics** | **Population n = 157** | **Mortality = 63** | **Survival = 94** | **p value** |  |
|  | **Reason for cardiogenic shock, n(%)** |  |  |  | 0.301 |  |
|  | Arrhythmic storm | 5 (3.5) | 2 (3.4) | 3 (3.5) |  |  |
|  | Postcardiotomy | 52 (36.4) | 20 (34.5) | 32 (37.6) |  |  |
|  | Pulmonary thromboembolism | 12 (8.4) | 5 (8.6) | 7 (8.2) |  |  |
|  | Acute coronary syndrome | 35 (24.5) | 15 (25.9) | 20 (23.5) |  |  |
|  | Decompensated heart failure | 15 (10.5) | 5 (8.6) | 10 (11.8) |  |  |
|  | Myocarditis | 11 (7.7) | 2 (3.4) | 9 (10.6) |  |  |
|  | Other | 13 (9.1) | 9 (15.5) | 4 (4.7) |  |  |
|  | *p <0,05 |  |  |  |  |  |
|  |  |  |  |  |  |  |
|  |  |  |  |  |  |  |
|  |  |  |  |  |  |  |
|  |  |  |  |  |  |  |
|  | **Supplementary Table 2. Other surgical events with cardiogenic shock and VA-ECMO** | | | | |  |
|  | **Characteristics** | **Population n = 157** | **Mortality = 63** | **Survival = 94** | **p value** |  |
|  | Myocardial revascularisation | 5 (3.5) | 2 (3.4) | 3 (3.5) | 0.668 |  |
|  | Pulmonary thrombectomy | 9 (5.7) | 2 (3.2) | 7 (7.4) | 0.259 |  |
|  | Aortic valve replacement | 18 (11.5) | 9 (14.3) | 9 (9.6) | 0.364 |  |
|  | Heart transplant | 6 (3.8) | 2 (3.2) | 4 (4.3) | 0.729 |  |
|  | Emergency surgery | 12 (8.4) | 3 (5.2) | 9 (10.6) | 0.251 |  |
|  | Mitral valve replacement | 20 (12.7) | 9 (14.3) | 11 (11.7) | 0.634 |  |
|  | Tricuspid valve replacement | 7 (4.5) | 4 (6.4) | 3 (3.2) | 0.347 |  |
|  | *p <0,05 |  |  |  |  |  |
|  |  |  |  |  |  |  |

**Supplementary Table S3.** Score composition and operational definitions (SOFA, APACHE II, SAVE) and DEOx formula

S3A. SOFA (Vincent et al., 1998) — domains, variables, thresholds, scoring (0–4 each)

| **Organ system** | **Variable (units)** | **Definition / thresholds** | **Score** |
| --- | --- | --- | --- |
| Respiratory | PaO₂/FiO₂ (mmHg) | ≥400 (0);  <400 (1);  <300 (2);  <200 **with respiratory support** (3);  <100 **with respiratory support** (4) | 0–4 |
| Coagulation | Platelets (×10⁹/L) | ≥150 (0);  <150 (1);  <100 (2);  <50 (3);  <20 (4) | 0–4 |
| Liver | Bilirubin (mg/dL) | <1.2 (0);  1.2–1.9 (1);  2.0–5.9 (2);  6.0–11.9 (3);  ≥12.0 (4) | 0–4 |
| Cardiovascular | MAP/vasopressors | 0: MAP ≥70 without vasopressors; 1: MAP <70;  2: dopamine ≤5 or dobutamine any;  3: dopamine >5 or epinephrine ≤0.1 or norepinephrine ≤0.1 μg·kg⁻¹·min⁻¹;  4: dopamine >15 or epinephrine >0.1 or norepinephrine >0.1 μg·kg⁻¹·min⁻¹ | 0–4 |
| CNS | Glasgow Coma Scale | 15 (0);  13–14 (1);  10–12 (2);  6–9 (3);  <6 (4) | 0–4 |
| Renal | Creatinine (mg/dL) **or** urine output | Cr <1.2 (0);  1.2–1.9 (1);  2.0–3.4 (2);  3.5–4.9 **or** urine <500 mL/day (3);  ≥5.0 **or** urine <200 mL/day (4) | 0–4 |

**Timing rule:** worst value within first **24 h post-cannulation.**
**Handling:** if an item is missing, use the closest clinically justifiable value within the window and document in the Case Report Form (CRF).

S3B. APACHE II (Knaus et al., 1985) — components and scoring

| **Component** | **Variables included** | **Notes on scoring** | **Points** |
| --- | --- | --- | --- |
| Acute Physiology Score (APS) | 12 variables: Temperature; Mean arterial pressure; Heart rate; Respiratory rate; Oxygenation (A-a gradient if FiO₂ ≥0.5, otherwise PaO₂); Arterial pH (or HCO₃⁻ if no ABG); Serum Na⁺; Serum K⁺; Serum creatinine (×2 if acute renal failure); Hematocrit; WBC count; GCS | Points per deviation from normal as per Knaus 1985 tables | contributes to total |
| Age points | Age categories per Knaus 1985 | Added to APS | 0–6 |
| Chronic health points | Severe organ insufficiency/immunocompromise | Added if criteria met | 0–5 |
| **Total APACHE II** | APS + Age + Chronic health | Per original publication | **0–71** |

**Timing rule:** worst values in first **24 h post-cannulation**; use pre-sedation GCS when available.

S3C. SAVE (Survival After Veno-Arterial ECMO) score — components and point assignments (Schmidt et al., 2015)

| **Parameter** | **Category / definition** | **Points** |
| --- | --- | --- |
| **Acute cardiogenic shock diagnosis group** (select one) | Myocarditis | +3 |
|  | Refractory ventricular tachycardia/fibrillation (VT/VF) | +2 |
|  | Post heart or lung transplantation | −3 |
|  | Congenital heart disease | −3 |
|  | Other diagnoses leading to cardiogenic shock requiring VA-ECMO | 0 |
| **Age (years)** | 18–38 | +7 |
|  | 39–52 | +3 |
|  | 53–62 | 0 |
|  | ≥63 | 0 |
| **Weight (kg)** | ≤65 | +1 |
|  | 65–89 | +2 |
|  | ≥90 | 0 |
| **Acute pre-ECMO organ failures** (select one or more if present) | Liver failureᵃ | −3 |
|  | Central nervous system dysfunctionᵇ | −3 |
|  | Renal failureᶜ | −3 |
|  | Chronic renal failureᵈ | −6 |
| **Duration of intubation prior to ECMO (h)** | ≤10 | 0 |
|  | 11–29 | −2 |
|  | ≥30 | −4 |
| **Physiology prior to ECMO (binary items)** | Peak inspiratory pressure ≤20 cmH₂O | +3 |
|  | Pre-ECMO cardiac arrest | −2 |
|  | Diastolic blood pressure before ECMO ≥40 mmHgᵉ | +3 |
|  | Pulse pressure before ECMO ≤20 mmHgᵉ | −2 |
|  | HCO₃⁻ before ECMO ≤15 mmol/L | −3 |
| **Constant** | Add to all calculations | **+6** |
| **Total SAVE score (after adding constant)** | Possible range | **−35 to +23** |

| **Total SAVE score** | **Risk class** | **Estimated survival (%)** |
| --- | --- | --- |
| >5 | I | 75 |
| 1–5 | II | 58 |
| −4 to 0 | III | 42 |
| −9 to −5 | IV | 30 |
| ≤−10 | V | 18 |

**Footnotes / operational definitions**
ᵃ Liver failure: bilirubin ≥33 µmol/L or ALT/AST >70 U/L.
ᵇ CNS dysfunction: neurotrauma, stroke, encephalopathy, cerebral metastasis, seizures/epileptic syndromes.
ᶜ Renal failure: chronic or acute renal insufficiency (e.g., creatinine >1.5 mg/dL) with or without RRT.
ᵈ Chronic renal failure (CKD): kidney damage or eGFR <60 mL/min/1.73 m² for ≥3 months.
ᵉ Timing for BP indices: use the **worst value within 6 hours prior to ECMO cannulation**

**Computation note:** SAVE predicts **survival**; for comparability we transformed survival to **mortality** (see Methods and Table 6).
**Timing:** variables taken per original SAVE definition; worst values within **24 h** where applicable.

S3D. DEOx — formula and operationalization

| **Item** | **Definition** |
| --- | --- |
| Formula | **DEOx = 6.322 × Lactate − 2.311 × Base excess − 9.013** |
| Inputs | Arterial lactate (mmol/L) and base excess (mmol/L) |
| Units | mL O₂/kg (indirect estimate) |
| Timing | First **24 h post-cannulation**, worst value used |
| Rationale | Reflects early **metabolic debt** (transition to anaerobic metabolism) |

S3E. Cut-offs and operating characteristics used in Table 6

| **Score/metric** | **Cut-off** | **Se (%)** | **Sp (%)** | **LR+** | **LR−** | **AUROC (95% CI)** |
| --- | --- | --- | --- | --- | --- | --- |
| APACHE II | ≥12 | 54.1 | 55.2 | 1.21 | 0.83 | 0.611 (0.51–0.71) |
| SOFA | ≥6 | 63.9 | 38.6 | 1.04 | 0.93 | 0.595 (0.49–0.69) |
| SAVE (mortality) | <−2 | 75.4 | 20.2 | 0.94 | 1.21 | 0.625 (from survival) |
| DEOx | ≥3.78 | 64.7 | 56.3 | 1.48 | 0.63 | 0.663 (0.49–0.77) |

**Scoring window for all systems:** worst values within **24 h** after cannulation.

**Supplementary Checklist S1.** STROBE Statement-- Checklist of items that should be included in reports of ***cohort studies***

|  | Item No | Recommendation |
| --- | --- | --- |
| **Title and abstract** | 1 | (*a*) Indicate the study’s design with a commonly used term in the title or the abstract |
|  |  | (*b*) Provide in the abstract an informative and balanced summary of what was done and what was found |
| Introduction | | |
| Background/rationale | 2 | Explain the scientific background and rationale for the investigation being reported |
| Objectives | 3 | State specific objectives, including any prespecified hypotheses |
| Methods | | |
| Study design | 4 | Present key elements of study design early in the paper |
| Setting | 5 | Describe the setting, locations, and relevant dates, including periods of recruitment, exposure, follow-up, and data collection |
| Participants | 6 | (*a*) Give the eligibility criteria, and the sources and methods of selection of participants. Describe methods of follow-up |
|  |  | (*b*) For matched studies, give matching criteria and number of exposed and unexposed |
| Variables | 7 | Clearly define all outcomes, exposures, predictors, potential confounders, and effect modifiers. Give diagnostic criteria, if applicable |
| Data sources/ measurement | 8* | For each variable of interest, give sources of data and details of methods of assessment (measurement). Describe comparability of assessment methods if there is more than one group |
| Bias | 9 | Describe any efforts to address potential sources of bias |
| Study size | 10 | Explain how the study size was arrived at |
| Quantitative variables | 11 | Explain how quantitative variables were handled in the analyses. If applicable, describe which groupings were chosen and why |
| Statistical methods | 12 | (*a*) Describe all statistical methods, including those used to control for confounding |
|  |  | (*b*) Describe any methods used to examine subgroups and interactions |
|  |  | (*c*) Explain how missing data were addressed |
|  |  | (*d*) If applicable, explain how loss to follow-up was addressed |
|  |  | (*e*) Describe any sensitivity analyses |
| Results | | |
| Participants | 13* | (a) Report numbers of individuals at each stage of study—eg numbers potentially eligible, examined for eligibility, confirmed eligible, included in the study, completing follow-up, and analysed |
|  |  | (b) Give reasons for non-participation at each stage |
|  |  | (c) Consider use of a flow diagram |
| Descriptive data | 14* | (a) Give characteristics of study participants (eg demographic, clinical, social) and information on exposures and potential confounders |
|  |  | (b) Indicate number of participants with missing data for each variable of interest |
|  |  | (c) Summarise follow-up time (eg, average and total amount) |
| Outcome data | 15* | Report numbers of outcome events or summary measures over time |
| Main results | 16 | (*a*) Give unadjusted estimates and, if applicable, confounder-adjusted estimates and their precision (eg, 95% confidence interval). Make clear which confounders were adjusted for and why they were included |
|  |  | (*b*) Report category boundaries when continuous variables were categorized |
|  |  | (*c*) If relevant, consider translating estimates of relative risk into absolute risk for a meaningful time period |
| Other analyses | 17 | Report other analyses done—eg analyses of subgroups and interactions, and sensitivity analyses |
| Discussion | | |
| Key results | 18 | Summarise key results with reference to study objectives |
| Limitations | 19 | Discuss limitations of the study, taking into account sources of potential bias or imprecision. Discuss both direction and magnitude of any potential bias |
| Interpretation | 20 | Give a cautious overall interpretation of results considering objectives, limitations, multiplicity of analyses, results from similar studies, and other relevant evidence |
| Generalisability | 21 | Discuss the generalisability (external validity) of the study results |
| Other information | | |
| Funding | 22 | Give the source of funding and the role of the funders for the present study and, if applicable, for the original study on which the present article is based |

*Give information separately for exposed and unexposed groups.

**Note:** An Explanation and Elaboration article discusses each checklist item and gives methodological background and published examples of transparent reporting. The STROBE checklist is best used in conjunction with this article (freely available on the Web sites of PLoS Medicine at http://www.plosmedicine.org/, Annals of Internal Medicine at http://www.annals.org/, and Epidemiology at http://www.epidem.com/). Information on the STROBE Initiative is available at http://www.strobe-statement.org.
